# Functions of Rhotekin, an Effector of Rho GTPase, and Its Binding Partners in Mammals

**DOI:** 10.3390/ijms19072121

**Published:** 2018-07-20

**Authors:** Hidenori Ito, Rika Morishita, Koh-ichi Nagata

**Affiliations:** 1 Department of Molecular Neurobiology, Institute for Developmental Research, Aichi Human Service Center, 713-8 Kamiya, Kasugai 480-0392, Japan; itohide@inst-hsc.jp (H.I.); rmorishita@inst-hsc.jp (R.M.); 2 Department of Neurochemistry, Nagoya University Graduate School of Medicine, 65 Tsurumai-cho, Showa-ku, Nagoya 466-8550, Japan

**Keywords:** Rho, Rhotekin, binding partner

## Abstract

Rhotekin is an effector protein for small GTPase Rho. This protein consists of a Rho binding domain (RBD), a pleckstrin homology (PH) domain, two proline-rich regions and a C-terminal PDZ (PSD-95, Discs-large, and ZO-1)-binding motif. We, and other groups, have identified various binding partners for Rhotekin and carried out biochemical and cell biological characterization. However, the physiological functions of Rhotekin, per se, are as of yet largely unknown. In this review, we summarize known features of Rhotekin and its binding partners in neuronal tissues and cancer cells.

## 1. Introduction

**Figure 1:**
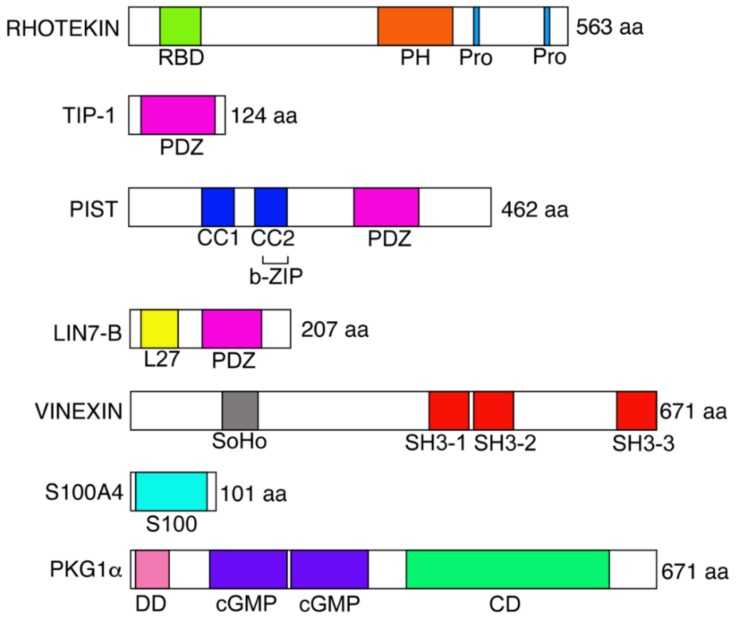
Structures of Rhotekin and its binding partners. Structural domains of proteins are abbreviated as follows: RBD, Rho-binding domain; PH, pleckstrin-homology; Pro, proline-rich motif; CC, coiled-coil domain; b-ZIP, basic leucine zipper domain; L27, Lin-2 and Lin-7 domain; PDZ, PSD-95/Discs large/Zona occludens-1 domain; SoHo, sorbin homologous domain; SH3, src homology 3 domain; S100, S100 domain; DD, dimerization/docking domain; cGMP, cyclic GMP binding domain; and, CD, catalytic domain.

The small GTPase Rho plays important roles in various cellular processes, including: Actin cytoskeletal reorganization; cell morphology; cell motility and tumor cell invasion; transcriptional regulation; and, cytokinesis [1,2]. In neuronal cells, Rho has been reported to regulate cellular processes such as neurite retraction and neuronal polarization [3,4]. Around 30 Rho effector molecules have been identified to date and have been shown to play pivotal roles in the neuronal functions [5]. Rhotekin was originally identified as a binding partner for Rho by yeast two-hybrid screening using a mouse embryo cDNA library [6]. The name is derived from the Japanese “teki”, meaning a target. While expression of Rhotekin mRNA was observed in the brain, kidney, lung and skeletal muscles [6], the protein is highly expressed in the brain; based on Western blot analyses [7,8]. Another family member, Rhotekin-2, has been found in lymphocyte [9]. Rhotekin has a predicted molecular mass of 61 kDa and contains characteristic domains and motifs such as: Active Rho-binding domain (RBD); Pleckstrin-homology (PH) domain; and, two Pro-rich motifs towards the C-terminus (Figure 1). In addition, Rhotekin exhibits at the C-terminus the sequence SPV-COOH that matches the X(S/T)XV-COOH consensus motif recognizing class I PDZ (PSD-95, Discs-large, and ZO-1)domains. The PDZ domain, an initialism combining the first letters of PSD(postsynaptic density protein)-95, Discs-large, and ZO(zonula occludens)-1, which is present in various proteins with diverse functions [10]. Many proteins binding to the PDZ domain are involved in the regulation of cell morphology, migration and signaling. The PH domain is known to bind with phosphatidylinositol 3,4,5-trisphosphate; a second messenger regulating the number of cellular processes [11], while the Pro-rich motif recognizes the Src-homology 3 (SH3) domain, a small conserved sequence of about 60 amino acid residues [12]. Thus, it is suggested that Rhotekin physiologically interacts with lipid membranes PDZ- and SH3-containing proteins. On the other hand, the RBD of Rhotekin selectively interacts with GTP-bound active Rho, and this domain inhibits both intrinsic and GTPase-activating protein (GAP)-enhanced GTPase activity of Rho [6]. Using these features of Rhotekin-RBD, the recombinant protein has been utilized for monitoring the activated state of Rho [13].

Among various potential effector proteins interacting with activated Rho, physiological functions have been revealed in some molecules, such as Rho-kinase/ROCK(Rho-associated, coiled-coil-containing protein kinase) and mDia(mouse Diaphanous), through intensive investigations. In contrast, the information about the function of Rhotekin is, as of yet, very limited. In this review, we summarize physiological features of Rhotekin and its binding partners.

## 2. Rho Family of Small GTPases

**Figure 2:**
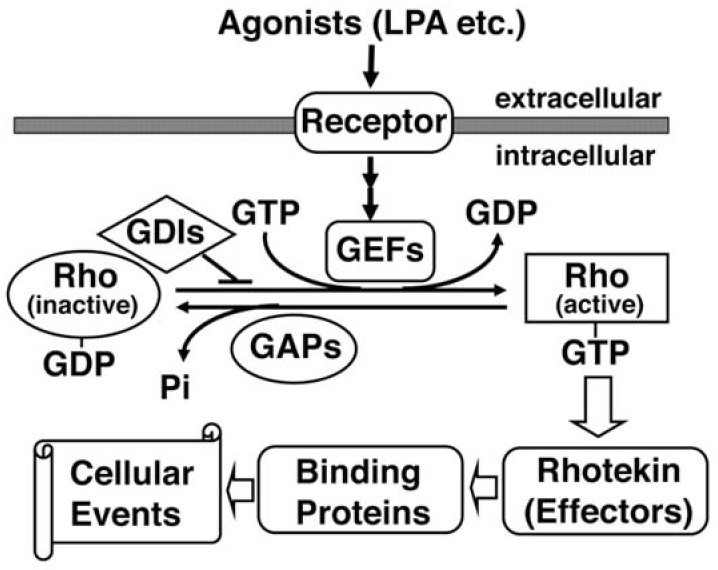
The supposed up and down-stream signaling paths of Rhotekin. Extracellular agonists such as lysophosphatidic acid (LPA) bind with a heterotrimeric G-protein-coupled receptor and activate specific GEFs (Guanine nucleotide exchange factors). GDI (GDP-dissociation inhibitor) selectively binds with the GDP-bound form of Rho and prevents the exchange of GDP for GTP. GEF catalyzes the exchange-bound GDP for GTP. The GTP-bound active Rho can interact with Rhotekin and may regulate the interaction between Rhotekin and its binding partners. GAP (GTPase activating protein) increases the intrinsic GTPase activity of Rho and causes the inactivation of the protein. Rhotekin may control various cellular events such as the proliferation of neural progenitor cells, the neurite outgrowth and the synapse formation.

Rho GTPases are members of the Ras superfamily of monomeric small GTPases with molecular masses of 20–30 kDa. Ten different mammalian Rho GTPase members have been identified: Rho; Rac; Cdc42; Rnd1/Rho6; Rnd2/Rho7; Rnd3/RhoE; RhoD; RhoG; TC10; and, TTF [14]. The most extensively characterized members are Rho, Rac and Cdc42. Like other small GTPases, Rho proteins act as a molecular switch that cycles between GTP-bound (active) and GDP-bound (inactive) states, which are regulated by three groups of regulatory proteins (Figure 2). Guanine nucleotide exchange factor (GEF) catalyzes the exchange of GDP for GTP, leading to activation in response to various upstream signals. GTPase-activating protein (GAP) increases the intrinsic GTPase activity, resulting in the inactivation of the protein. Finally, GDP-dissociation inhibitor (GDI) has the ability to block the cycling between the GDP and GTP-bound forms by preventing the exchange of GDP for GTP.

## 3. Rhotekin in Neuronal Tissues

**Figure 3:**
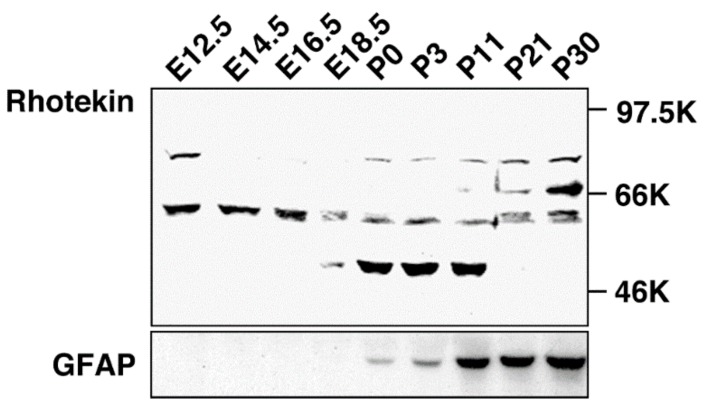
Developmental change of Rhotekin in the rat brain. Cholate extracts of brains at various stages were subjected to SDS-PAGE followed by Western blotting with anti-Rhotekin (**top**) or anti-GFAP (**bottom**). Molecular markers are at right. (Adapted from Reference [7]).

Rho family GTPases are known to regulate neuronal functions including axon guidance, dendrite formation and spine morphogenesis [3,4,5]. Cdc42 and Rac signaling pathways facilitate neurite formation while Rho-mediated signaling takes part in neurite retraction in cultured neuronal cells. While mRNA expression of Rhotekin has been analyzed [6], information about Rhotekin protein distribution in mammalian tissues, including its distribution in the brain, was lacking because the specific antibody for this protein had not been developed. Therefore, we produced a specific antibody, anti-Rhotekin, with its N-terminus 334 amino acids as an antigen [15]. Using anti-Rhotekin, we found a unique localization pattern of Rhotekin in rat brain during the developmental stage [7,8]. In Western blotting, anti-Rhotekin detected four major bands (molecular masses about 75 kDa, 68 kDa, 61 kDa and 50 kDa), which were considered splicing isoforms in rat brain at various developmental stages (Figure 3). The protein with 75 kDa was detected at embryonic day (E12.5 but disappeared until after birth and re-increased up to postnatal day P30. Another band with 68 kDa was only observed at P21 and P30. The 61 kDa protein was highly expressed at embryonic stages but gradually decreased during development. The 50 kDa band was detected at limited periods, E18.5 to P11. Immunofluorescence analysis revealed that Rhotekin is distributed in soma, axon and the dendrite of primary-cultured rat hippocampal neurons [7,8]. In dendrite, localization of Rhotekin partially overlapped with synaptophysin, a representative marker for synapse. These results suggest that Rhotekin may play various roles in neuronal development with isoform specific manners.

Thereafter, Iwai et al. reported that Rhotekin is required for the maintenance and survival of neurons and positively regulates the differentiation and neurite outgrowth in vitro [16]. They also found that Rhotekin is produced in neural stem cells (NSCs) and negatively regulates cell proliferation, suggesting that Rhotekin is one of the key molecules in the differentiation of NSCs into neurons. Taken together, Rhotekin may be one essential molecule for the development of neuronal tissues. However, the precise molecular mechanism of its function is obscure.

## 4. Rhotekin and Cancer Cells

Based on the physiological function of Rho in cell morphology, migration and growth, abnormal up-regulation of Rho signaling can possibly contribute to the invasion and metastasis of cancer cells. Indeed, there are several reports describing the abnormal expression of Rhotekin in cancer cells. Liu et al., reported that 71% of gastric cancer specimens examined overexpressed Rhotekin [17]. They also showed that the transfection of Rhotekin in AGS gastric cancer cells conferred the resistance to apoptosis induced by serum deprivation and treatment with sodium butyrate [18]. Rhotekin overexpression resulted in the activation of the nuclear kappa B (NF-κB) while inhibitors for NF-κB, curcumin or parthenolide, diminished the anti-apoptotic effect of Rhotekin [18]. These results suggest that Rho/Rhotekin-mediated NF-κB activation may play a key role in gastric tumorigenesis. Enhanced expression of Rhotekin was also detected in other cancer cells. By screening human colorectal carcinoma cell lines with two-dimensional gel electrophoresis, Rhotekin was identified as a metastasis-related protein [19]. In colon cancer, Rhotekin2 has also been reported to be overexpressed in surgically resected specimens and in two cell lines, SW480 and HCT116. Knockdown of *RTKN2* in these cell lines led to the notable inhibition of cell proliferation and cell cycle progression by reducing the expression of cell cycle-associated proteins such as Cyclin D1 and c-myc [20]. In human bladder carcinoma, mRNA levels of Rhotekin were significantly higher in surgically resected specimens than in tumor-free bladder tissues, suggesting that Rhotekin takes part in bladder carcinogenesis and the carcinoma progression [21]. In addition, Rhotekin expression levels were significantly higher in lung cancer patient samples compared to that of the adjacent normal tissues [22]. Analyses with lung adenocarcinoma cell lines (A549 and SPC-A-1) revealed that Rhotekin-knockdown reduces cell viability; probably due to S phase arrest and subsequent cell apoptosis, and suppresses their invasion activities [22]. Furthermore, the pathophysiological role of Rhotekin was investigated in hepatocellular carcinoma (HCC), the most common adult liver cancer, which explains approximately 90% of all cases of primary liver cancer annually. Rhotekin-mRNA was significantly increased in surgical specimens of HCC [23]. The investigation of the overexpression and silencing of Rhotekin in HCC cell lines, HepG2 and Hep3B, revealed that Rhotekin may function as an oncogene by promoting the proliferation and inhibiting apoptosis of these cells [23].

Taken together, Rhotekin-mediated signaling pathways may be a novel therapeutic target for cancer. In this context, recent studies have reported that micro RNAs (miRNAs), miRNA-152 [23] and let-7a [24] are likely to inhibit tumor cell growth through the down-regulation of Rhotekin expression.

## 5. Regulation of the Septin Cytoskeletal Organization by Rhotekin

Septins are a conserved family of cytoskeletal GTP/GDP-binding proteins and are present in organisms as diverse as yeast and mammals [25,26]. Genetic and cell biological examinations indicate their essential roles in cytokinesis, cell cycle, plasma membrane compartmentalization, vesicle trafficking and the regulation of some signal transduction pathways [27]. Thirteen Septin genes (*Sept1*–*Sept14*, *Sept13* is a pseudogene) have been identified in humans. They form the filamentous structure in mammalian cells [28], but the information about the regulatory mechanisms of the Septin complex is limited. However, Borg, a Cdc42 effector, is suggested to be a regulator of Septin organization [29].

It is notable that Septin gene abnormalities have been reported to associate with tumorgenesis [30]. In this context, Rho/Rhotekin signaling pathways have been shown to regulate the structure of Septin filaments [15]. The activated form of Rho disrupted the filament structure in rat embryonic fibroblast REF52 cells. Transfection of Rhotekin resulted in the similar morphological change of Septin filaments. Further analyses revealed that the center region of Rhotekin was essential for the interaction and the transfection of this region, which caused disruption of the Septin filaments. These results suggest that Rhotekin is an important linker between Rho signaling and Septin complexes.

## 6. Identification and Characterization of Binding Partners for Rhotekin

**Figure 4:**
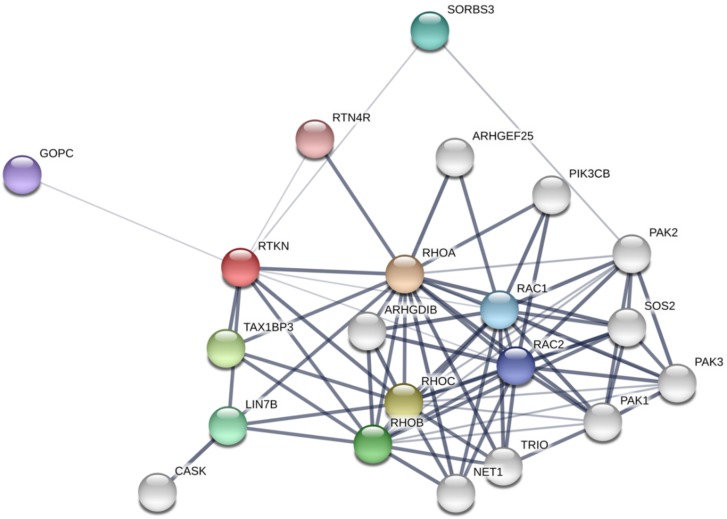
Rhotekin-interacting protein network analysis using STRING software. The thickness of the line indicates the strength of the interaction. Note that the interaction between Rhotekin (RTKN) and Rac has contradicted a previous report [6]. Colored nodes, the query protein and first shell of interactors; White nodes, second shell of interactors.

A number of online resources and databases are now available to understand protein-protein interaction networks. For example, STRING database [31] depicts the network of Rhotekin-binding proteins as shown in Figure 4. On the other hand, we have identified PIST (GOPC, Golgi-associated PDZ and coiled-coil motif-containing protein; FIG, fused in glioblastoma; CAL, CFTR-associated ligand), LIN-7 (Veli, MALS) and vinexin (SORBS3) as binding partners for Rhotekin by using yeast two-hybrid analysis with a human heart cDNA library [8,32,33]. In this part, we summarize characteristics of several binding partners for Rhotekin.

### 6.1. TIP-1 (TAX1BP3)

TIP (Tax-interaction protein)-1 (TAX1BP3) was identified as a binding partner for Tax oncoprotein of T-lymphotropic virus type 1 (HTLV-1) [34]. TIP-1 is a cytoplasmic protein with 124 amino acids that have a single PDZ domain (Figure 1). When Reynaud et al., conducted a yeast two-hybrid analysis using TIP-1 as bait, they identified Rhotekin as an interacting protein [35]. In the same study, they revealed that the C-terminus PDZ-binding motif of Rhotekin was responsible for the binding to the PDZ domain in TIP-1. In addition, transient expression analyses with a reporter construct, including the c-Fos serum response element (SRE), indicated that Rhotekin activates TIP-1 in a Rho-dependent manner and causes a strong activation of the SRE [35]. It is notable that a dominant negative Rho was unable to cooperate with TIP-1 and Rhotekin, and TIP-1 lost the above positive effects when the interaction with Rhotekin was impaired. The data shows that the cytoplasmic ternary complex of active Rho/Rhotekin/TIP-1 plays an essential role in triggering the activation of the SRE within the nucleus—leading to transcriptional regulation.

### 6.2. PIST (GOPC, FIG, CAL)

PIST, also called GOPC, FIG or CAL, was first identified as a putative effector protein for TC10 [36,37,38]. PIST contains two putative coiled-coil domains, a basic Leu-zipper within the second coiled-coil domain and a PDZ domain (Figure 1). PIST was shown to be localized at Golgi-apparatus in several cultured cell lines, maybe through its interaction with the Q-SNARE (Q-soluble NSF attachment protein receptor) protein syntaxin-6 [36]. The second coiled-coil domain was shown to be important for PIST localization at the golgi-apparatus [36]. The Leu-zipper appeared to be essential for the interaction with activated TC10 [37].

In addition to the Golgi-apparatus, PIST was shown to bind to the cell surface proteins frizzled five and frizzled eight, CALEB (chloride channel ClC-3B and chicken acidic leucine-rich epidermal growth factor-like domain-containing brain protein), neuroglycan C, CFTR (cystic fibrosis transmembrane conductance regulator), and, to regulate the expression of these proteins at the plasma membrane [38,39,40,41]. Moreover, PIST interacts with the β1-adrenergic receptor and regulates the trafficking of the receptor in the biosynthetic pathway and during postendocytic recycling [42]. PIST may also stabilize the β1-adrenergic receptor in intracellular compartments after internalization [42]. From these observations, PIST could possibly be localized at both Golgi-apparatus and plasma membranes. In this context, we have recently demonstrated the localization of PIST at the Golgi-apparatus in non-polarized epithelial MDCK (Madin-Darby canine kidney) cells and adherens junctions (AJs) in the polarized cells, indicating that PIST may be recruited from the cytosol to AJs during cell polarization [32]. We also clarified that Rho-signal activation or prevention of Rhotekin-PIST interaction induced diffuse cytoplasmic distribution of Rhotekin in polarized MDCK cells. Despite the fact that the precise mechanism of the translocation of PIST and Rhotekin remains to be elucidated, Rho may regulate Rhotekin-PIST interaction and PIST may play an essential role in the recruitment of Rhotekin to AJs.PIST was reported to participate in vesicle transport between Golgi and plasma membranes in non-neuronal cells [36,38,39,40,41]. Meanwhile, a neuron-specific isoform, neuronal PIST (nPIST), which differs from PIST by an eighth amino acid insertion within the second coiled-coil domain, is localized in neuronal synapses. nPIST plays a pivotal role in AMPA (α-amino-3-hydroxy-5-methylisoxazole-4-propionic acid) receptor trafficking to synapses through the interaction with stargazin, an AMPA receptor-interacting protein [43]. nPIST plays roles in autophagy and neurodegeneration through the interaction with delta 2 glutamate receptor (GluR δ2) and Beclin [44]. From these results, PIST most likely takes part in intracellular vesicle and/or protein transport in a variety of cells.

Despite the molecular mechanism regulating PIST functions being almost unknown, our finding of PIST-Rhotekin interaction may become a clue to clarify the mechanism. We demonstrated interaction between Rhotekin and PIST by in vitro pull-down assays and immunoprecipitation assays using MDCK cells and rat brain tissues [7,32]. We also showed that active Rho suppresses PIST-Rhotekin interaction, whereas inhibition of Rho by C3 exoenzyme had little effect on the interaction. In contrast, activated TC10, a possible upstream regulator of PIST, had no effects on the interaction. These results suggest that PIST-Rhotekin interaction is regulated by Rho signals, although PIST function(s) may be controlled by TC10. While Rhotekin and PIST were colocalized at the cell–cell contact sites of epithelial cells as mentioned above, they were colocalized with a presynaptic marker, synaptophysin, in primary cultured rat hippocampal neurons in immunofluorescence analyses. It should be noted that synapses are the sites of communication between neurons and their targets, and should be recognized as specialized variants of the cell-cell junctions. On the other hand, a novel role of PIST has been suggested at cell-cell contact sites since PIST forms immunocomplex with another PDZ-binding protein, β-catenin, in the rat brain. It is notable that PIST was colocalized with β-catenin at dendritic filopods in rat primary cultured hippocampal neurons [7]. Rhotekin and β-catenin are mutually antagonistic in their interaction with PIST in vitro; immunocomplex formation of PIST with β-catenin was suppressed by co-expression of Rhotekin in HEK293 cells. These results suggest the yet unidentified role of Rhotekin, which may be in harmony with PIST and β-catenin, in regulation of cell-cell junction formation and maintenance in both neuronal and non-neuronal cells. The interaction of PIST with β-catenin may also regulate cell polarity through the formation and maintenance of cell-cell junctions.

While Rhotekin and PIST are downstream effectors for Rho and TC10 respectively, Rho, but not TC10, was found to affect the Rhotekin-PIST interaction. The balance between complexes of Rhotekin-PIST and β-catenin-PIST controlled by Rho activity may be important for PIST and/or β-catenin functions. To elucidate the physiological role of the complex formation of PIST with Rhotekin or β-catenin during cell polarization and in cell signaling, further extensive analyses will be required.

The interaction of Rhotekin with PIST implies the involvement of Rhotekin in PIST-mediated protein trafficking in synapses, autophagy and neurodegeneration, as mentioned above. Interestingly, Rhotekin and PIST are expressed in developmental stage-specific manners in the brain [7], suggesting that the mode of their interaction may change during the developmental stages and the PIST-Rhotekin complex may have various roles during the neuronal development.

### 6.3. LIN-7 (Veli, MALS)

Proper targeting of proteins at the apical or basolateral plasma membranes of epithelial cells is important for intracellular transport. Likewise, proper targeting of proteins to axons or dendrites is essential for neurotransmission. Indeed, many studies have focused on the molecular mechanisms that determine the direction of protein sorting. In this context, PDZ domain-containing proteins are now recognized as pivotal players in this evolutionarily conserved pathway. LIN-7 (also called Veli and MALS) is a small scaffold protein identified as a mammalian homolog of *Caenorhabditis elegans* LIN-7 [45], which contains an L27 domain and a PDZ domain (Figure 1). There are three mammalian homologs of LIN-7, LIN7-A, -B, and -C [46,47]. In *C. elegans*, LIN-7 forms a heterotrimeric complex with two other PDZ proteins, LIN-2 and LIN-10, and the complex ensures the localization of the LET-23 growth factor receptor of the lateral/junctional domain of vulval precursor cells, which is required to keep the signaling cascade active for the proliferation and differentiation of vulval cells [48,49,50]. A corresponding complex of LIN-2, LIN-7, and LIN-10 has been identified in mammalian brain [46,51]. The mammalian homologues of LIN-2 and LIN-10 are known as CASK and Mint1/X11, respectively [49,51,52,53]. In neurons, it is possible to bind the ternary complexes to specific target membrane sites via Munc-18-1, a protein that modulates vesicle trafficking and secretion [54]. Once bound to membranes, LIN-2, LIN-7 and LIN-10 may serve to localize or retain proteins at specific membrane sites, either individually or as a complex form. LIN-2/CASK have been detected at neuronal synapses [55] as well as at the basolateral surface of epithelial cells [52]. LIN-7 and Rhotekin were also partially colocalized at the synapses of primary cultured rat hippocampal neurons and in cell-cell contact in polarized MDCK cells [8]. Taken together, the LIN-7-Rhotekin complex is likely to be important for the cell polarity formation and/or maintenance.

LIN-7 appears to be involved in other synapse functions since it associates with the neuronal cadherin-β-catenin complex [56] and the PSD-95/NMDA receptor complex [8,46,47]. In the course of investigating Rhotekin function in neuronal tissues, we identified LIN-7 as its interactive partner [8]. The C-terminal PDZ-binding motif was responsible for the binding to LIN-7. We found that both LIN-7 and Rhotekin are abundant in the brain in Western blot analyses. Immunohistochemical analyses then revealed that LIN-7 distributes in neurons at the thalamus, hippocampus CA1 region and dentate gyrus. It is notable that this localization profile was similar to that of Rhotekin. We then demonstrated that Rhotekin interacts with LIN-7B in vitro and forms immunocomplex in brain tissues. Interestingly, active Rho facilitated LIN-7-Rhotekin interaction, suggesting that their interaction is regulated by Rho signals and that the Rho/Rhotekin signal affects the function of LIN-7. In the case of PIST, Rhotekin may exert a novel function through interaction with LIN-7. It is tempting to speculate that LIN-7 and Rhotekin are involved in various neuronal functions in a concerted manner. While the molecular mechanisms remain to be elucidated, interaction of Rhotekin with LIN-7 also suggests that Rhotekin plays a role in the function and/or regulation of LIN-2/LIN-7/LIN-10 complex in synapses. Further intensive analyses are required to elucidate this issue.

We, and others, have reported the involvement of LIN-7 in neurodevelopmental disease, such as attention deficit hyperactivity disorder (ADHD) [57,58] and autism [59]. We assume that Rhotekin may play a number of important roles in the regulation of synaptic transmission in concert with LIN-7.

### 6.4. Vinexin (SORBS3)

Vinexin (SORBS3) is a member of a multi-domain adaptor family, which was originally identified as a binding partner for vinculin [60]. There are three isoforms in mammals: Vinexin-α, -β; -γ. A; and, β isoforms, which have sorbin-homology (SoHo) domain and three SH3 domains (Figure 1) while γ isoform is a short variant that contains only three SH3 domains. Vinexin is thought to be involved in the actin cytoskeletal organization and cellular processes such as migration, spreading and proliferation [61]. Vinexin is localized in the synapses of primary cultured hippocampal neurons and is phosphorylated by ERK (Extracellular signal-regulated kinase) [62]. We have identified vinexin as a binding partner for Rhotkein using yeast two-hybrid analysis [33]. While the Pro-rich motif at C-terminus of Rhotekin interacts with the third SH3 domain of vinexin, the interaction was independent of Rho activity. Rhotekin and vinexin were at least partially colocalized at focal adhesions of REF52 fibroblast cells.

Recently, Chang and Huang reported the role of Rhotekin and vinexin in the cell cycle progression [63]. They showed the colocalization of Rhotekin and vinexin at the midbody during cell division. Overexpression of vinexin mutant, lacking the Rhotekin-binding motif and knockdown of vinexin or Rhotekin in HeLa cells, caused the increase of cells arrested at the midbody stage. They concluded that vinexin localized at the midbody recruits Rhotekin to facilitate the cytokinesis.

It has been suggested that Vinexin is involved in pathophysiology of atherosclerosis [64] and cardiac hypertrophy [65]. Suppression of Akt–NF-κB signaling pathways are likely to underlie these disease conditions. On the other hand, vinexin-β has also been reported to act as a modulator of ischemic injury. Deletion of vinexin-β potently protected against ischemic injury by inhibiting neuronal apoptosis, which may occur via the up-regulation of Akt–NF-κB signaling [66]. The precise molecular function of Rho and Rhotekin in the pathogenicity of these disorders remain to be clarified.

### 6.5. S100 Calcium-Binding Protein A4 (S100A4)

S100A4 is a member of the S100 calcium-binding protein superfamily, which includes more than 20 family members [67,68]. S100A4 contains two EF-hands calcium-binding motifs and is considered to associate with the progression of various cancers such as: Colon; breast; prostate; gallbladder; gastric; lung; and melanoma [69,70,71,72,73,74]. Recently, Chen et al., reported that S100A4 selectively interacts with Rhotekin-RBD but not with RBDs of other Rho effectors such as Citron, mDia and Rho-kinase/ROCK [75]. S100A4 and Rhotekin are colocalized at the leading edge of migrating cells. S100A4 binds to Rhotekin in a calcium-dependent manner, and S100A4, Rhotekin and active RhoA, form a tripartite complex [75]. From the RNA interference experiments, it is suggested that S100A4 and Rhotekin co-operate to inhibit actin stress fiber formation and promote membrane ruffle formation induced by epidermal growth factor (EGF) stimulation. From these results, S100A4-Rhotekin interaction appears to control the Rho signaling outcome by spatially affecting the Rho-mediated actin cytoskeleton assembling and modification. Consequently, S100A4 and Rhotekin are most likely to contribute to the conferring of invasive phenotypes of cancer cells.

### 6.6. Cyclic GMP-Dependent Kinase (PKG or cGK)

Cyclic GMP is known to regulate neurite outgrowth and neuronal migration [76,77,78]. Cyclic GMP-dependent kinase (PKG or cGK) is one of the central targets of cGMP. Two types of PKG, PKG1 and PKG2, are encoded by different genes in mammals. Two isoforms of PKG1, PKG1α and PKG1β, exist in mammals. Yuasa et al., identified Rhotekin as an interacting molecule for PKG1α [79]. Subsequent analyses revealed that PKG1β more strongly interacted with Rhotekin. Notably, PKG1α mediates GMP-dependent phosphorylation of Rhotekin at Ser93, which lowered the affinity between the two molecules in vitro. Treatment with a membrane-permeable cGMP analog (8-pCPT-cGMP) decreased the interaction between PKG1 and Rhotekin, transiently expressed in COS7 cells. While Rhotekin and PKG1α are localized at the plasma membrane and extended neurites in Neuro2A neuroblast cells, Rhotekin is translocated to the cytoplasm upon 8-pCPT-cGMP treatment. In neurons, Rho negatively regulates neurite outgrowth and Rho-kinase/ROCK mediates Rho-dependent neurite retraction [80]. Lysophosphatidic acid (LPA) is known to retract neurites of neurons via the activation of Rho [81]. LPA-induced neurite retraction was suppressed by cGMP/PKG1α through the phosphorylation of Rhotekin [79], indicating that Rhotekin and PKG1α may contribute to the Rho-mediated neurite outgrowth regulation.

Deregulation of PKG1α has been demonstrated to be involved in many cardiovascular diseases [82]. While the molecular mechanisms underlying these disorders are not fully understood, the association of PKG1α to Rho-Rhotekin signals might be a clue to elucidate the pathophysiology of the abovementioned disorders.

## 7. Concluding Remarks

**Figure 5:**
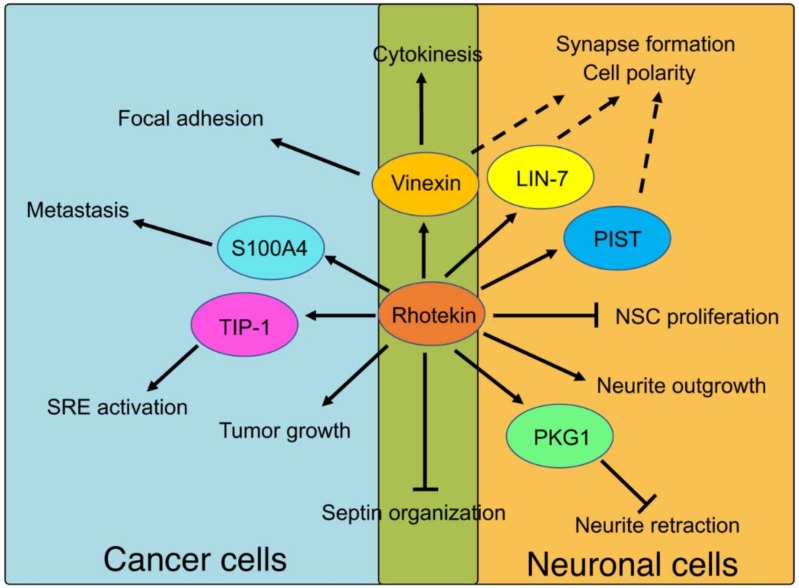
Physiological roles of Rhotekin and its binding partners. Solid lines represent events that are fully understood. Broken lines represent predicted roles that remain to be clarified. The abbreviations used are as follow; NSC, neural stem cells; SRE, serum response element.

Possible roles of Rhotekin and its binding partners in cancer and neuronal cells are summarized in Figure 5. We have reported a possible involvement of Rhotekin in cell polarity, neuronal synapse formation and focal adhesion [7,8,32,33,62]. However, precise molecular mechanisms underlying these processes are, currently, largely unknown. Further intensive studies with various experimental approaches are required to address this issue. Since Rhotekin is abundantly expressed in brain tissues and in several kinds of cancer cells, the investigation of Rhotekin function may also contribute to the understanding of the pathophysiology of neurodevelopmental disorder and cancer.

## Abbreviations

RBD	Rho binding domain
PH	pleckstrin homology
GAP	GTPase activating protein
GEF	guanine nucleotide exchange factor
GDI	GDP-dissociation inhibitor
NF-κB	nuclear kappa B
HCC	hepatocellular carcinoma
MDCK	Madin-Darby canine kidney
AJ	adherence junctions
PKG	cyclic GMP dependent kinase
SH3	Src homology 3
NSC	neural stem cells
SRE	serum response element

## References

[1] Etienne-Manneville S., Hall A. (2002). Rho GTPases in cell biology. Nature.

[2] Jaffe A.B., Hall A. (2002). Rho GTPases in transformation and metastasis. Adv. Cancer Res..

[3] Govek E.E., Newey S.E., Van Aelst L. (2005). The role of the Rho GTPases in neuronal development. Genes Dev..

[4] Negishi M., Katoh H. (2002). Rho family GTPases as key regulators for neuronal network formation. J. Biochem. (Tokyo).

[5] Bito H. (2003). Dynamic control of neuronal morphogenesis by rho signaling. J. Biochem. (Tokyo).

[6] Reid T., Furuyashiki T., Ishizaki T., Watanabe G., Watanabe N., Fujisawa K., Morii N., Madaule P., Narumiya S. (1996). Rhotekin, a new putative target for Rho bearing homology to a serine/threonine kinase, PKN, and rhophilin in the rho-binding domain. J. Biol. Chem..

[7] Ito H., Iwamoto I., Mizutani K., Morishita R., Deguchi T., Nozawa Y., Asano T., Nagata K. (2006). Possible interaction of a Rho effector, Rhotekin, with a PDZ-protein, PIST, at synapses of hippocampal neurons. Neurosci. Res..

[8] Sudo K., Ito H., Iwamoto I., Morishita R., Asano T., Nagata K. (2006). Identification of a cell polarity-related protein, Lin-7B, as a binding partner for a Rho effector, Rhotekin, and their possible interaction in neurons. Neurosci. Res..

[9] Collier F.M., Gregorio-King C.C., Gough T.J., Talbot C.D., Walder K., Kirkland M.A. (2004). Identification and characterization of a lymphocytic Rho-GTPase effector: Rhotekin-2. Biochem. Biophys. Res. Commun..

[10] Sheng M., Sala C. (2001). PDZ domains and the organization of supramolecular complexes. Annu. Rev. Neurosci..

[11] Lemmon M.A., Ferguson K.M. (2000). Signal-dependent membrane targeting by pleckstrin homology (PH) domains. Biochem. J..

[12] Li S.S. (2005). Specificity and versatility of SH3 and other proline-recognition domains: Structural basis and implications for cellular signal transduction. Biochem. J..

[13] Ren X.D., Kiosses W.B., Schwartz M.A. (1999). Regulation of the small GTP-binding protein Rho by cell adhesion and the cytoskeleton. EMBO J..

[14] Bishop A.L., Hall A. (2000). Rho GTPases and their effector proteins. Biochem. J..

[15] Ito H., Iwamoto I., Morishita R., Nozawa Y., Narumiya S., Asano T., Nagata K. (2005). Possible role of Rho/Rhotekin signaling in mammalian septin organization. Oncogene.

[16] Iwai T., Saitoh A., Yamada M., Takahashi K., Hashimoto E., Ukai W., Saito T. (2012). Rhotekin modulates differentiation of cultured neural stem cells to neurons. J. Neurosci. Res..

[17] Liu C.A., Wang M.J., Chi C.W., Wu C.W., Chen J.Y. (2004). Overexpression of rho effector rhotekin confers increased survival in gastric adenocarcinoma. J. Biomed. Sci..

[18] Liu C.A., Wang M.J., Chi C.W., Wu C.W., Chen J.Y. (2004). Rho/Rhotekin-mediated NF-kappaB activation confers resistance to apoptosis. Oncogene.

[19] Ying-Tao Z., Yi-Ping G., Lu-Sheng S., Yi-Li W. (2005). Proteomic analysis of differentially expressed proteins between metastatic and non-metastatic human colorectal carcinoma cell lines. Eur. J. Gastroenterol. Hepatol..

[20] Pang X., Li R., Shi D., Pan X., Ma C., Zhang G., Mu C., Chen W. (2017). Knockdown of Rhotekin 2 expression suppresses proliferation and induces apoptosis in colon cancer cells. Oncol. Lett..

[21] Fan J., Ma L.J., Xia S.J., Yu L., Fu Q., Wu C.Q., Huang X.H., Jiang J.M., Tang X.D. (2005). Association between clinical characteristics and expression abundance of RTKN gene in human bladder carcinoma tissues from Chinese patients. J. Cancer Res. Clin. Oncol..

[22] Zhang W., Liang Z., Li J. (2016). Inhibition of rhotekin exhibits antitumor effects in lung cancer cells. Oncol. Rep..

[23] Zhou J., Zhang Y., Qi Y., Yu D., Shao Q., Liang J. (2017). MicroRNA-152 inhibits tumor cell growth by directly targeting RTKN in hepatocellular carcinoma. Oncol. Rep..

[24] Li B., Chen P., Chang Y., Qi J., Fu H., Guo H. (2016). Let-7a inhibits tumor cell growth and metastasis by directly targeting RTKN in human colon cancer. Biochem. Biophys. Res. Commun..

[25] Field C.M., Kellogg D. (1999). Septins: Cytoskeletal polymers or signalling GTPases?. Trends Cell Biol..

[26] Trimble W.S. (1999). Septins: A highly conserved family of membrane-associated GTPases with functions in cell division and beyond. J. Membr. Biol..

[27] Dolat L., Hu Q., Spiliotis E.T. (2014). Septin functions in organ system physiology and pathology. Biol. Chem..

[28] Kinoshita M. (2003). Assembly of mammalian septins. J. Biochem. (Tokyo).

[29] Joberty G., Perlungher R.R., Sheffield P.J., Kinoshita M., Noda M., Haystead T., Macara I.G. (2001). Borg proteins control septin organization and are negatively regulated by Cdc42. Nat. Cell Biol..

[30] Angelis D., Spiliotis E.T. (2016). Septin Mutations in Human Cancers. Front. Cell Dev. Biol..

[31] Szklarczyk D., Morris J.H., Cook H., Kuhn M., Wyder S., Simonovic M., Santos A., Doncheva N.T., Roth A., Bork P. (2017). The STRING database in 2017: Quality-controlled protein-protein association networks, made broadly accessible. Nucleic Acids Res..

[32] Ito H., Iwamoto I., Morishita R., Nozawa Y., Asano T., Nagata K. (2006). Identification of a PDZ protein, PIST, as a binding partner for Rho effector Rhotekin: Biochemical and cell-biological characterization of Rhotekin-PIST interaction. Biochem. J..

[33] Nagata K., Ito H., Iwamoto I., Morishita R., Asano T. (2009). Interaction of a multi-domain adaptor protein, vinexin, with a Rho-effector, Rhotekin. Med. Mol. Morphol..

[34] Rousset R., Fabre S., Desbois C., Bantignies F., Jalinot P. (1998). The C-terminus of the HTLV-1 Tax oncoprotein mediates interaction with the PDZ domain of cellular proteins. Oncogene.

[35] Reynaud C., Fabre S., Jalinot P. (2000). The PDZ protein TIP-1 interacts with the Rho effector rhotekin and is involved in Rho signaling to the serum response element. J. Biol. Chem..

[36] Charest A., Lane K., McMahon K., Housman D.E. (2001). Association of a novel PDZ domain-containing peripheral Golgi protein with the Q-SNARE (Q-soluble N-ethylmaleimide-sensitive fusion protein (NSF) attachment protein receptor) protein syntaxin 6. J. Biol. Chem..

[37] Neudauer C.L., Joberty G., Macara I.G. (2001). PIST: A novel PDZ/coiled-coil domain binding partner for the rho-family GTPase TC10. Biochem. Biophys. Res. Commun..

[38] Yao R., Maeda T., Takada S., Noda T. (2001). Identification of a PDZ domain containing Golgi protein, GOPC, as an interaction partner of frizzled. Biochem. Biophys. Res. Commun..

[39] Cheng J., Moyer B.D., Milewski M., Loffing J., Ikeda M., Mickle J.E., Cutting G.R., Li M., Stanton B.A., Guggino W.B. (2002). A Golgi-associated PDZ domain protein modulates cystic fibrosis transmembrane regulator plasma membrane expression. J. Biol. Chem..

[40] Gentzsch M., Cui L., Mengos A., Chang X.B., Chen J.H., Riordan J.R. (2003). The PDZ-binding chloride channel ClC-3B localizes to the Golgi and associates with cystic fibrosis transmembrane conductance regulator-interacting PDZ proteins. J. Biol. Chem..

[41] Hassel B., Schreff M., Stube E.M., Blaich U., Schumacher S. (2003). CALEB/NGC interacts with the Golgi-associated protein PIST. J. Biol. Chem..

[42] Koliwer J., Park M., Bauch C., von Zastrow M., Kreienkamp H.J. (2015). The golgi-associated PDZ domain protein PIST/GOPC stabilizes the beta1-adrenergic receptor in intracellular compartments after internalization. J. Biol. Chem..

[43] Cuadra A.E., Kuo S.H., Kawasaki Y., Bredt D.S., Chetkovich D.M. (2004). AMPA receptor synaptic targeting regulated by stargazin interactions with the Golgi-resident PDZ protein nPIST. J. Neurosci..

[44] Yue Z., Horton A., Bravin M., DeJager P.L., Selimi F., Heintz N. (2002). A novel protein complex linking the delta 2 glutamate receptor and autophagy: Implications for neurodegeneration in lurcher mice. Neuron.

[45] Irie M., Hata Y., Deguchi M., Ide N., Hirao K., Yao I., Nishioka H., Takai Y. (1999). Isolation and characterization of mammalian homologues of Caenorhabditis elegans lin-7: Localization at cell-cell junctions. Oncogene.

[46] Butz S., Okamoto M., Südhof T.C. (1998). A Tripartite Protein Complex with the Potential to Couple Synaptic Vesicle Exocytosis to Cell Adhesion in Brain. Cell.

[47] Jo K., Derin R., Li M., Bredt D.S. (1999). Characterization of MALS/Velis-1, -2, and -3: A Family of Mammalian LIN-7 Homologs Enriched at Brain Synapses in Association with the Postsynaptic Density-95/NMDA Receptor Postsynaptic Complex. J. Neurosci..

[48] Hoskins R., Hajnal A.F., Harp S.A., Kim S.K. (1996). The *C. elegans* vulval induction gene lin-2 encodes a member of the MAGUK family of cell junction proteins. Development.

[49] Kaech S.M., Whitfield C.W., Kim S.K. (1998). The LIN-2/LIN-7/LIN-10 Complex Mediates Basolateral Membrane Localization of the *C. elegans* EGF Receptor LET-23 in Vulval Epithelial Cells. Cell.

[50] Simske J.S., Kaech S.M., Harp S.A., Kim S.K. (1996). LET-23 Receptor Localization by the Cell Junction Protein LIN-7 during *C. elegans* Vulval Induction. Cell.

[51] Borg J.-P., Straight S.W., Kaech S.M., de Taddéo-Borg M., Kroon D.E., Karnak D., Turner R.S., Kim S.K., Margolis B. (1998). Identification of an Evolutionarily Conserved Heterotrimeric Protein Complex Involved in Protein Targeting. J. Biol. Chem..

[52] Cohen A.R., Wood D.F., Marfatia S.M., Walther Z., Chishti A.H., Anderson J.M. (1998). Human CASK/LIN-2 Binds Syndecan-2 and Protein 4.1 and Localizes to the Basolateral Membrane of Epithelial Cells. J. Cell Biol..

[53] Hata Y., Butz S., Sudhof T.C. (1996). CASK: A novel dlg/PSD95 homolog with an N-terminal calmodulin-dependent protein kinase domain identified by interaction with neurexins. J. Neurosci..

[54] Okamoto M., Südhof T.C. (1997). Mints, Munc18-interacting Proteins in Synaptic Vesicle Exocytosis. J. Biol. Chem..

[55] Hsueh Y.-P., Yang F.-C., Kharazia V., Naisbitt S., Cohen A.R., Weinberg R.J., Sheng M. (1998). Direct Interaction of CASK/LIN-2 and Syndecan Heparan Sulfate Proteoglycan and Their Overlapping Distribution in Neuronal Synapses. J. Cell Biol..

[56] Perego C., Vanoni C., Massari S., Longhi R., Pietrini G. (2000). Mammalian LIN-7 PDZ proteins associate with β-catenin at the cell–cell junctions of epithelia and neurons. EMBO J..

[57] Lanktree M., Squassina A., Krinsky M., Strauss J., Jain U., Macciardi F., Kennedy J.L., Muglia P. (2008). Association study of brain-derived neurotrophic factor (BDNF) and LIN-7 homolog (LIN-7) genes with adult attention-deficit/hyperactivity disorder. Am. J. Med. Genet. B.

[58] Shinawi M., Sahoo T., Maranda B., Skinner S.A., Skinner C., Chinault C., Zascavage R., Peters S.U., Patel A., Stevenson R.E. (2011). 11p14.1 microdeletions associated with ADHD, autism, developmental delay, and obesity. Am. J. Med. Genet. A.

[59] Mizuno M., Matsumoto A., Hamada N., Ito H., Miyauchi A., Jimbo E.F., Momoi M.Y., Tabata H., Yamagata T., Nagata K. (2015). Role of an adaptor protein Lin-7B in brain development: Possible involvement in autism spectrum disorders. J. Neurochem..

[60] Kioka N., Sakata S., Kawauchi T., Amachi T., Akiyama S.K., Okazaki K., Yaen C., Yamada K.M., Aota S. (1999). Vinexin: A novel vinculin-binding protein with multiple SH3 domains enhances actin cytoskeletal organization. J. Cell Biol..

[61] Kioka N., Ueda K., Amachi T. (2002). Vinexin, CAP/ponsin, ArgBP2: A novel adaptor protein family regulating cytoskeletal organization and signal transduction. Cell Struct. Funct..

[62] Ito H., Usuda N., Atsuzawa K., Iwamoto I., Sudo K., Katoh-Semba R., Mizutani K., Morishita R., Deguchi T., Nozawa Y. (2007). Phosphorylation by extracellular signal-regulated kinase of a multidomain adaptor protein, vinexin, at synapses. J. Neurochem..

[63] Chang Y.W., Huang Y.S. (2017). Midbody localization of vinexin recruits rhotekin to facilitate cytokinetic abscission. Cell Cycle.

[64] Guan H., Cheng W.L., Guo J., Chao M.L., Zhang Y., Gong J., Zhu X.Y., She Z.G., Huang Z., Li H. (2017). Vinexin β Ablation Inhibits Atherosclerosis in Apolipoprotein E–Deficient Mice by Inactivating the Akt–Nuclear Factor κB Inflammatory Axis. J. Am. Heart Assoc..

[65] Liu X., Wan N., Zhang X.-J., Zhao Y., Zhang Y., Hu G., Wan F., Zhang R., Zhu X., Xia H. (2015). Vinexin-β exacerbates cardiac dysfunction post-myocardial infarction via mediating apoptotic and inflammatory responses. Clin. Sci..

[66] Li M., Guo S., Zhang P., Gong J., Zheng A., Zhang Y., Li H. (2015). Vinexin-beta deficiency protects against cerebral ischaemia/reperfusion injury by inhibiting neuronal apoptosis. J. Neurochem..

[67] Marenholz I., Lovering R.C., Heizmann C.W. (2006). An update of the S100 nomenclature. Biochim. Biophys. Acta.

[68] Santamaria-Kisiel L., Rintala-Dempsey A.C., Shaw G.S. (2006). Calcium-dependent and -independent interactions of the S100 protein family. Biochem. J..

[69] Gupta S., Hussain T., MacLennan G.T., Fu P., Patel J., Mukhtar H. (2003). Differential expression of S100A2 and S100A4 during progression of human prostate adenocarcinoma. J. Clin. Oncol..

[70] Kimura K., Endo Y., Yonemura Y., Heizmann C.W., Schafer B.W., Watanabe Y., Sasaki T. (2000). Clinical significance of S100A4 and E-cadherin-related adhesion molecules in non-small cell lung cancer. Int. J. Oncol..

[71] Maelandsmo G.M., Florenes V.A., Mellingsaeter T., Hovig E., Kerbel R.S., Fodstad O. (1997). Differential expression patterns of S100A2, S100A4 and S100A6 during progression of human malignant melanoma. Int. J. Cancer.

[72] Rudland P.S., Platt-Higgins A., Renshaw C., West C.R., Winstanley J.H., Robertson L., Barraclough R. (2000). Prognostic significance of the metastasis-inducing protein S100A4 (p9Ka) in human breast cancer. Cancer Res..

[73] Takenaga K., Nakanishi H., Wada K., Suzuki M., Matsuzaki O., Matsuura A., Endo H. (1997). Increased expression of S100A4, a metastasis-associated gene, in human colorectal adenocarcinomas. Clin. Cancer Res..

[74] Yonemura Y., Endou Y., Kimura K., Fushida S., Bandou E., Taniguchi K., Kinoshita K., Ninomiya I., Sugiyama K., Heizmann C.W. (2000). Inverse expression of S100A4 and E-cadherin is associated with metastatic potential in gastric cancer. Clin. Cancer Res..

[75] Chen M., Bresnick A.R., O’Connor K.L. (2013). Coupling S100A4 to Rhotekin alters Rho signaling output in breast cancer cells. Oncogene.

[76] Yamazaki M., Chiba K., Mohri T. (2005). Fundamental role of nitric oxide in neuritogenesis of PC12h cells. Br. J. Pharmacol..

[77] Murray A.J., Peace A.G., Shewan D.A. (2009). cGMP promotes neurite outgrowth and growth cone turning and improves axon regeneration on spinal cord tissue in combination with cAMP. Brain Res..

[78] Zhao Z., Wang Z., Gu Y., Feil R., Hofmann F., Ma L. (2009). Regulate axon branching by the cyclic GMP pathway via inhibition of glycogen synthase kinase 3 in dorsal root ganglion sensory neurons. J. Neurosci..

[79] Yuasa K., Nagame T., Dohi M., Yanagita Y., Yamagami S., Nagahama M., Tsuji A. (2012). cGMP-dependent protein kinase I is involved in neurite outgrowth via a Rho effector, rhotekin, in Neuro2A neuroblastoma cells. Biochem. Biophys. Res. Commun..

[80] Hirose M., Ishizaki T., Watanabe N., Uehata M., Kranenburg O., Moolenaar W.H., Matsumura F., Maekawa M., Bito H., Narumiya S. (1998). Molecular Dissection of the Rho-associated Protein Kinase (p160ROCK)-regulated Neurite Remodeling in Neuroblastoma N1E-115 Cells. J. Cell Biol..

[81] Jalink K., van Corven E.J., Hengeveld T., Morii N., Narumiya S., Moolenaar W.H. (1994). Inhibition of lysophosphatidate- and thrombin-induced neurite retraction and neuronal cell rounding by ADP ribosylation of the small GTP-binding protein Rho. J. Cell Biol..

[82] Lee D.I., Kass D.A. (2012). Phosphodiesterases and cyclic GMP regulation in heart muscle. Physiology (Bethesda).

